# Frequency of first and second-line drug resistance-associated mutations among resistant *Mycobacterium tuberculosis* clinical isolates from São Paulo, Brazil

**DOI:** 10.1590/0074-02760200055

**Published:** 2020-05-08

**Authors:** Tania Matsui, Juliana Maíra Watanabe Pinhata, Michelle Christiane da Silva Rabello, Angela Pires Brandão, Lucilaine Ferrazoli, Sylvia Cardoso Leão, Cristina Viana-Niero, Rosangela Siqueira de Oliveira

**Affiliations:** 1 Universidade Federal de São Paulo, Departamento de Microbiologia, Imunologia e Parasitologia, São Paulo, SP, Brasil; 2 Instituto Adolfo Lutz, Centro de Bacteriologia, Núcleo de Tuberculose e Micobacterioses, São Paulo, SP, Brasil; 3 Fundação Oswaldo Cruz-Fiocruz, Instituto Aggeu Magalhães, Departamento de Imunologia, Recife, PE, Brasil

**Keywords:** MDR-TB, pre-XDR-TB, XDR-TB, *gyr*A, *gyr*B, rrs

## Abstract

**BACKGROUND:**

Tuberculosis (TB) is an infectious disease caused by the bacterium *Mycobacterium tuberculosis*, and the number of new cases of multidrug resistant TB (MDR-TB), pre extensively drug-resistant TB (pre-XDR-TB) and extensively drug-resistant TB (XDR-TB) has increased considerably worldwide.

**OBJECTIVES:**

Herein, using 156 *M. tuberculosis* isolates from 106 patients previously classified as MDR or pre-XDR or XDR isolates, we investigated the genetic mutation profiles associated with phenotypic resistances in patients with MDR-TB, pre-XDR-TB and XDR-TB, treatment outcomes and resistance evolution.

**METHODS:**

Molecular analyses were performed by partial sequencing of the *rpo*B, *kat*G, *gyr*A, *gyr*B, *rrs* genes and analysis of the *fab*G-*inh*A promoter region. Clinical, epidemiologic and demographic data were obtained from the TB Notification database system of São Paulo (TB-WEB) and the Information System for Special Tuberculosis Treatments (SITE-TB).

**FINDINGS:**

Drug resistance was attributed to previously known mutations and a novel Asp449Val mutation in *gyr*B was observed in four isolates from the same patient. Ten patients had more than one isolate evaluated and eight of these patients displayed resistance progression.

**MAIN CONCLUSIONS:**

The present study is the first to report the frequency of mutations related to second-line drug resistance in MDR-TB, pre-XDR-TB and XDR-TB isolates. The results could lead to the improvement of available technologies for the rapid detection of drug resistant TB.

Tuberculosis (TB) is an infectious disease caused by the bacterium *Mycobacterium tuberculosis*. While most cases can be treated with antibiotics, drug resistance is one of the main challenges for TB control and a leading cause of death related to this disease.^1^ It is well known that TB drug resistance is mainly conferred by specific point mutations in the *M. tuberculosis* genome.^2^ ^,^ ^3^ ^,^ ^4^ ^,^ ^5^ ^,^ ^6^ Multidrug-resistant TB (MDR-TB), which is caused by *M. tuberculosis* isolates with resistance to isoniazid (INH) and rifampicin (RIF), is the main threat to disease control and elimination.^1^ ^,^ ^7^ Additionally, pre-extensively drug-resistant TB (pre-XDR-TB) is characterised by additional resistance to any fluoroquinolone (FQ) drug or at least one of the three second-line injectable drugs (SLID), and XDR-TB is defined as MDR *M. tuberculosis* with resistance to both FQ and a second-line injectable drug.^7^

In 2018, the World Health Organization (WHO) reported that there were an estimated 390,000 new cases of MDR-TB worldwide, with 548 new cases of TB with RIF resistance/MDR in Brazil alone.^1^ ^,^ ^8^ In 2017, the Ministry of Health of Brazil initiated the National Plan for the end of TB with the goal of reducing the 90% incidence and 95% mortality rates by 2035. One of the proposed strategies included molecular tests for drug resistance detection, which would allow for a more rapid diagnosis.^9^

There are few studies in Brazil reporting the frequency of mutations found in the genes associated with *M. tuberculosis* resistance to first- (INH, RIF, ethambutol - EMB, pyrazinamide - PZA, and streptomycin - SM) and second-line drugs [aminoglycosides (amikacin - AMK, kanamycin - KAN), the cyclic peptide capreomycin - CAP, and FQs (levofloxacin - LFX and ofloxacin - OFX)].^7^ ^,^ ^10^ ^,^ ^11^ ^,^ ^12^ ^,^ ^13^ Such data, using molecular techniques for the diagnosis of drug resistance in *M. tuberculosis*, could facilitate the development of algorithms that rapidly, efficiently and accurately diagnose patients with drug-resistant TB. Thus, the aim of the present study was to examine the frequency and distribution of genetic mutations in *M. tuberculosis* isolates from Brazilian patients in the state of São Paulo with drug-resistant TB, that confer resistance to INH, RIF, aminoglycosides and FQs. In addition, we also assessed the treatment outcomes and evolution of bacterial resistance.

## SUBJECTS AND METHODS

*Clinical isolates* - A total of 156 *M. tuberculosis* isolates from the Reference Laboratory for Tuberculosis and Mycobacteriosis, Instituto Adolfo Lutz (IAL), in São Paulo, Brazil, were analysed. All of the isolates were previously characterised for drug susceptibility using the BD Bactec^TM^ MGIT^TM^ 960 automated system. From January 2016 to December 2017, 140 of the *M. tuberculosis* isolates from 106 patients were classified as either isoniazid resistant (n = 1); MDR (n = 107), pre-XDR (n = 21) or XDR (n = 11). Additionally, in order to evaluate the resistance profile over time, 16 isolates, belonging to 10 of the 106 patients and collected from 2012 to 2015, were also included in this study. These 10 isolates were classified as isoniazid resistant (n = 1); MDR (n = 6), pre-XDR (n = 6) or XDR (n = 3). This study was approved by the IAL Ethics Committee (Plataforma Brasil CAAE no. 78889517.9.1001.0059; Adolfo Lutz Institute nº 2.453.558).

*Data collection* - The drug susceptibility testing results were obtained from the Hospital Information and Management System at the IAL (SIGH-IAL) and the Laboratory Environment Management System (GAL) databases, and the demographic and clinical information were acquired from the online TB Notification database system of São Paulo (TB-WEB) and the Information System for Special Tuberculosis Treatments (SITE-TB).

Collected data included gender, ethnicity, age, education level, forms of TB presentation, HIV status, comorbidities (diabetes mellitus, smoking, alcohol abuse, drug addiction and others), TB history, treatment groups (new case, relapse and retreatment or lost to follow-up) and treatment outcomes. Patients were followed until October 2019 and outcomes were grouped as successful, which refers to patients who were cured; unsuccessful, which refers to patients who experienced treatment failure, were lost to follow-up or died regardless of the cause during treatment; or ongoing, which refers to patients who were still receiving treatment at the time of analysis.

*Sequencing of rpo*B*, kat*G*, gyr*A*, gyr*B*, and rrs genes and the fab*G*-inh*A *region* - The DNA of the bacterial isolates was extracted by thermal lysis. Briefly, 1 mL of each *M. tuberculosis* isolate cultured in Middlebrook 7H9 medium was supplemented with OADC (Oleic Acid, Albumin, Dextrose and Catalase) (BD BACTEC™ MGIT™ 960), incubated at 95ºC for 30 min, frozen at -20ºC for 2 h and was centrifuged at 13.000 x g for 1 min. Then, 5 µL of the supernatant was used for each polymerase chain reaction (PCR) reaction. The PCR-amplified regions included the RIF Resistance Determining Region (RRDR) of *rpo*B, the region containing codon 315 of *kat*G, the Quinolone Resistance Determining Regions (QRDR) of *gyr*A and *gyr*B, known resistance-associated regions of the *rrs* genes and the promoter region of *fab*G/*inh*A.^2^ ^,^ ^3^ ^,^ ^4^ ^,^ ^5^ ^,^ ^6^ The gene-specific primers used are listed in Supplementary data **(Table I)**. The negative controls consisted of all of the PCR reagents and components, but no DNA was added. The amplicons were purified using the QIAquick PCR purification kit (Qiagen, Germany), according to the recommendations of the manufacturer and the purified PCR products were sequenced in both directions in an ABI Prism 3500xL Sequencer (Applied Biosystems, Foster City, CA, USA). The same primers used for the PCR reactions were also employed for DNA sequencing.

To generate single consensus sequences, the nucleotide sequences were edited using the BioNumerics program version 7.6.3 (Applied Maths, Sint-Martens-Latem, Belgium), which were then compared with the *M. tuberculosis* H37Rv sequence deposited in the National Center for Biotechnology Information (NCBI) database under access number NC000962.3 (URL: http://www.ncbi.nlm.nih.gov/BLAST).

*Phenotypic and molecular drug susceptibility testing* - Isolates with discrepancies between the phenotypic and sequencing results were retested for drug susceptibility to first- (INH and RIF) and second-line drugs (LFX, AMK and KAN) as previously described.^14^ ^,^ ^15^ The identification of mutations that confer resistance to first- and second-line drugs was performed using line probe assays (LPA) GenoTypeMTBDR*plus* v2.0 and MTBDR*sl*v2.0 (HainLifescience, Nehren, Germany) with DNA from the isolates that was extracted from drug- and non-drug-containing MGIT tubes, and was performed according to instructions of the manufacturer. DNA extracted from the *M. tuberculosis* H37Rv strain was used as the positive control, and distilled water was used as the negative control.

*Heteroresistance analysis* - Isolates that showed hybridisation to all of the wild type (WT) probes, as well as those that hybridised to any representative mutation probes in the LPA assay, were then sequenced using the DNA extracted from cultures in the presence and absence of the respective drug and subsequently subjected to molecular typing to evaluate the existence of heteroresistance. For molecular typing, DNA was extracted and typed by IS*6110* restriction fragment length polymorphism (IS*6110*-RFLP) as described by van Embden et al.^16^ The RFLP patterns were analysed with the BioNumerics program version 7.6.3 (Applied Maths, Sint-Martens-Latem, Belgium), and the similarity of patterns was calculated using the Unweighted pair-group Methodwith Arithmetic mean (UPGMA) and the Dice similarity coefficient (1.5% of tolerance).

## RESULTS

As shown in Table I, among the 156 clinical isolates analysed, 152 (97.4%) presented *rpo*B RRDR mutations, which conferred RIF resistance and were consistent with the phenotypic data. No *rpo*B RRDR mutations were found in one isolate (0.65%) that presented a susceptible phenotype, thus showing agreement between the two methods. In contrast, three isolates (1.95%) presented discordant results between the phenotypic and molecular methods. For example, no mutations were observed in two RIF resistant isolates, and one isolate had a resistance mutation in the region but exhibited a phenotypically susceptible profile (Table I). With regard to INH resistance, 135 (86.5%) isolates showed concordant results in both methods. On the other hand, 21 (13.5%) isolates showed discordant results, with no mutations identified in *kat*G or *fab*G*-inh*A (Table I).

**TABLE I:** DNA sequencing of *rpo*B RRDR, *kat*G 315 codon and the *fab*G-*inh*A region and first-line drug susceptibility testing results

Sequencing/target	BD BACTEC^TM^ MGIT^TM^ 960			
	Rifampicin		Isoniazid	
	Susceptible N (%)	Resistant N (%)	Susceptible N (%)	Resistant N (%)
Presence of mutation in *rpo*B	1 (0.65)	152 (97.4)	NA	NA
Absence of mutation in *rpo*B	1 (0.65)	2 (1.3)	NA	NA
Presence of mutation in *kat*G	NA	NA	0	106 (67.9)
Presence of *fab*G-*inh*A mutation	NA	NA	0	20 (12.8)
Presence of mutation in *kat*G and *fab*G-*inh*A	NA	NA	0	9 (5.8)
Absence of mutation in *kat*G and *fab*G-*inh*A	NA	NA	0	21 (13.5)
Total	156 (100)		156 (100)	

NA: not applicable.

The mutation patterns detected in *rpo*B, *kat*G and *fab*G-*inh*A are summarised in Table II. Of the 152 isolates presenting mutations the *rpo*B RRDR region, 117 (77%) showed mutation at codon 531, which resulted in a serine to leucine (Ser531Leu) change in 109 of the isolates. Additionally, mutations at codons 516 and 526 were found in 15 (19.2%) of the isolates. In addition to the mutations identified at codon 531, three isolates had additional mutations at codons 490, 505 and 569 of the *rpo*B RRDR, and another mutation outside the RRDR was observed at codon 572 in one isolate. Notably, codons 517 and 518 were found to be deleted in two isolates.

Mutations in *kat*G were identified in 115 (73.7%) of the isolates analysed. In 112 (71.8%) isolates, mutations were found at codon 315 and the most frequently observed amino acid change was a serine to threonine (Ser315Thr) mutation, which was identified in 104 (92.9%) of these 112 isolates. Mutations were also detected in other codons, with three (1.9%) isolates exhibiting mutation at codons 259 and 381. In 29 (18.6%) isolates, mutations were found in the *fab*G-*inh*A, and 26 (89.7%) of these 29 isolates had the C-15T mutation and three (10.3%) had G-17T mutation (Table II).

**TABLE II:** Mutations identified in *rpo*B RRDR, *kat*G and the *fab*G-*inh*A region

Mutation in *rpo*B gene/ Codon and amino acid change	Mutation in *kat*G gene/ Codon and amino acid change	*fab*G-*inh*A region position and mutation	Number of isolates	%
Ser531Leu (TCG => TTG)	Ser315Thr (AGC => ACC)	NM	65	41.67
Ser531Leu (TCG => TTG)	NM	C -15 T	19	12.18
Ser531Leu (TCG => TTG)	NM	NM	15	9.62
Ser531Trp (TCG => TGG)	Ser315Thr (AGC => ACC)	NM	6	3.85
Ser531Leu (TCG => TTG)	Ser315Asn (AGC => AAC), Ala506Thr (GCC => ACC)	C -15 T	4	2.56
Ser531Trp (TCG => TGG)	Ser315Asn (AGC => AAC)	NM	2	1.28
Ser531Leu (TCG => TTG)	Asp259Gly (GAC => GGC)	NM	2	1.28
Ser531Leu (TCG => TTG)	Ser315Gly (AGC => GGC)	C -15 T	1	0.64
Ser531Leu (TCG => TTG)	Ser315Asn (AGC => AAC)	NM	1	0.64
Ser531Leu (TCG => TTG), Gln490Arg (CAG => CGG)	NM	C -15 T	1	0.64
Ser531Leu (TCG => TTG), Ile569Val (ATC => GTC)	Ser315Thr (AGC => ACC)	NM	1	0.64
His526Tyr (CAC => TAC)	Ser315Thr (AGC => ACC)	NM	4	2.56
His526Leu (CAC => CTC)	Ser315Thr (AGC => ACC)	G -17 T	3	1.92
His526Leu (CAC => CTC)	Ser315Thr (AGC => ACC)	NM	2	1.28
His526Cys (CAC => TGC)	Ser315Thr (AGC => ACC)	NM	2	1.28
His526Asp (CAC => GAC)	NM	NM	1	0.64
His526Asn (CAC => AAC)	NM	NM	1	0.64
His526Arg (CAC => CGC)	Ser315Thr (AGC => ACC)	NM	1	0.64
His526Asn (CAC => AAC), Phe505Leu (TTC => TTA)	Ser315Thr (AGC => ACC)	C -15 T	1	0.64
Asp516Val (GAC => GTC)	Ser315Thr (AGC => ACC)	NM	12	7.70
Asp516Tyr (GAC => TAC)	Ser315Thr (AGC => ACC)	NM	1	0.64
Asp516Tyr (GAC => TAC)	NM	NM	1	0.64
Asp516Ile (GAC => ATC)	Ser315Thr (AGC => ACC)	NM	1	0.64
Gln513Glu (CAA => GAA)	Ser315Thr (AGC => ACC)	NM	1	0.64
Ser522Leu (TCG => TTG)	Ser315Thr (AGC => ACC), Gly273Gly (GGT => GGC)	NM	1	0.64
Leu533Pro (CTG => CCG)	Ser315Thr (AGC => ACC)	NM	1	0.64
Ile572Tyr (ATC => TAC)	Ser315Thr (AGC => ACC)	NM	1	0.64
codon 518 deletion (AAC => del)	Asp381Asn (GAC => AAC)	NM	1	0.64
518 and 517 Codons deleted Asp516Asn (GAC => AAC) Met515Gln (ATG => CAG)	Ser315Thr (AGC => ACC)	NM	1	0.64
NM	NM	NM	3	1.92
Total			156	100,00

NM: no mutation; A: adenine; C: cytosine; G: guanine; T: thymine; Ala: alanine; Asn: asparagine; Arg: arginine; Asp: aspartic acid; Cys: cysteine; del: deletion; Gln: glutamine; Glu: glutamic acid; Gly: glycine; His: histidine; Ile: isoleucine; Leu: leucine; Phe: phenylalanine; Pro: proline; Ser: serine; Thr: threonine; Trp: tryptophan; Tyr: tyrosine; Val: valine.

Moreover, both the Ser531Leu mutation in the *rpo*B gene and the Ser315Thr mutation in the *kat*G gene were found to be present in 65 (41.67%) isolates, while 19 (12.18%) isolates were found to have both the Ser531Leu mutation in the *rpo*B gene and the C-15T mutation in the *fab*G-*inh*A region (Table II).

With regards to second-line drugs, 27 (17.3%) of the 156 isolates studied were phenotypically characterised as pre-XDR. Of these, 26 (96.3%) were FQ resistant and one (3.7%) was aminoglycoside resistant. Twenty-three (88.5%) of the 26 FQ resistant isolates had mutations in the QRDR of the *gyr*A gene, with 15 (57.7%) of these mutations present at codon 94. Additionally, a mutation in the QRDR of *gyr*B at codon 499 was observed in 7.7% (2/26) of the FQ resistant isolates, which results in an asparagine to aspartate (Asn499Asp) mutation. Two (3.8%) isolates showed discordant results between the phenotypic and molecular tests, with one being aminoglycoside resistant and the other FQ resistant; however, no mutations in the *rrs*, *gyr*A and *gyr*B genes that would confer antibiotic resistance, were detected (Table III).

Fourteen (9%) of the 156 isolates evaluated were found to be XDR, based on the phenotypic test, and all of these isolates presented mutations in the QRDR of *gyr*A and/or *gyr*B. In 71.4% (10/14) of these isolates, the A1401G mutation in the *rrs* gene was identified (Table III). Of these ten, four isolates presented an additional, previously undescribed, mutation in the *gyr*B gene that resulted in an aspartate to valine substitution (Asp449Val). In the remaining four isolates the phenotypic test indicated aminoglycoside resistance, yet no mutations in the *rrs* gene were detected.

**TABLE III:** Mutations identified in the *gyr*A, *gyr*B and *rrs* gene fragments of pre-XDR and XDR *Mycobacterium tuberculosis* isolates

Resistance profile	Mutations Codon and amino acid change			Number of isolates	%
	*gyr*A	*gyr*B	*rrs*				
Pre-XDR	Ala90Val (GCG => GTG),	NM	NM	4	14.8
	Ser91Pro (TCG => CCG),	NM	NM	4	14.8
	Asp94Gly (GAC => GGC),	NM	NM	11	40.7
	Asp94Asn (GAC => AAC),	NM	NM	3	11.1
	Asp94Asn (GAC => AAC),	Lys526Lys (AAG => AAA)	NM	1	3.7
	NM^#^	Asn499Asp (AAC => GAC)	NM	2	7.4
	NM^#^	NM	NM	2*	7.4
	Total			27	100
XDR	Gly88Ala (GGC => GCC)	NM	A1401G	1	7.1
	Asp94Gly (GAC => GGC),	NM	A1401G	5	35.7
	NM^#^	Asp449Val (GAT => GTT)	A1401G	4	28.6
	Asp94Gly (GAC => GGC, NM^#^	NM	NM	2	14.3
	NM^#^	Asn499Asp (AAC => GAC)	NM	2	14.3
	Total			14	100

*: one isolate aminoglycoside-resistant and one isolate FQ-resistant; ^#^: isolates with Ser95Thr polymorphism (does not confer resistance); NM: no mutation; A: adenine; C: cytosine; G: guanine; T: thymine; Ala: alanine; Asn: asparagine; Asp: aspartic acid; Gly: glycine; Pro: proline; Ser: serine; Thr: threonine; Val: valine.

The phenotypic results were confirmed after repeating the drug susceptibility test on all the isolates that exhibited discordant results with the first-line drugs (21 INH resistant and three RIF resistant) and second-line drugs (one LFX resistant and five aminoglycoside resistant).

To assess heteroresistance, isolates were cultured in drug-free tubes (control) and in tubes with drugs to be tested by reverse hybridisation in the GenoType MTBDR*plus* or GenoType MTBDR*sl* assays. Hybridisation of the MUT1 probe, which detects the C-15T mutation in the *fab*G-*inh*A region in one isolate and hybridisation with the MUT1 probe of *gyr*A (Ala90Val), were observed. For both isolates, the mutations were only detected in the drug-containing tubes. Moreover, these isolates were also analysed by IS*6110*-RFLP and showed the identical profiles of the isolates cultivated in the absence and presence of INH and LFX. These results indicate the existence of different subpopulations of the same *M. tuberculosis* strain (Figure).

**Figure:**
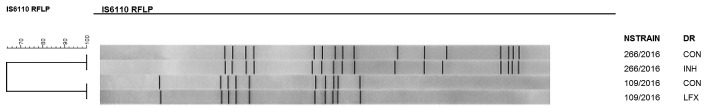
IS*6110*-RFLP patterns in two strains that exhibited heteroresistance. NStrain: number of strain; DR: drug resistance; CON: control; INH: isoniazid; LFX: levofloxacin.

As shown in Table IV, the socio-demographic profile and clinical characteristics of the 106 patients were analysed. In the MDR-TB group, the majority of patients were men (65/90; 72.2%), while in the pre-XDR-TB group and XDR-TB group the majority were women, 54.5% (6/11) and 60% (3/5), respectively.

In the three groups (MDR-TB, pre-XDR-TB and XDR-TB) the disease mainly affected patients with ages between 30-59 (59.6%, 63.6%, and 60%, respectively), The majority was HIV-negative (83.5%, 80% and 75%, respectively) and the dominant clinical form was pulmonary TB (95.4%, 100% and 100%, respectively). Regarding co-morbidities and risk behavior, smoking was the most frequent for MDR-TB (18.4%) followed by diabetes mellitus (11.5%). Notably, smoking and diabetes mellitus were also associated with other comorbidities.

Previous TB history was observed in 60.2% (53/90) of the MDR-TB patients, 54.5% (6/11) of the pre-XDR-TB patients and 80% (4/5) of the XDR-TB patients. Relapse was only observed in MDR-TB patients (21/95, 23.9%). In addition, retreatment was observed in 36.3% (32/90) of the MDR-TB patients, 54.5% (6/11) of the pre-XDR-TB patients and 80% (4/5) of the XDR-TB patients (Table IV).

**TABLE IV:** Socio-demographic profile and clinical characteristics of 106 patients with drug resistant tuberculosis from São Paulo State

Characteristics	MDR-TB (n = 90)				pre-XDR-TB (n = 11)				XDR-TB (n = 5)			
2016	2017	n	%	2016	2017	n	%	2016	2017	n	%
Gender (n = 106)												
Male	35	30	65	72.2	4	1	5	45.5	2	-	2	40.0
Female	12	13	25	27.8	5	1	6	54.5	2	1	3	60.0
Ethnicity (n = 99)												
White	16	19	35	42.2	3	1	4	36.4	3	1	4	80.0
Brown	21	14	35	42.2	4	1	5	45.5	1	-	1	20.0
Black	6	7	13	15.6	2	-	2	18.1	-	-	-	-
No information	4	3	7		-	-	-		-	-	-	
Age (years) (n = 105)												
15-29	11	10	21	23.6	3	-	3	27.3	-	1	1	20.0
30-59	31	22	53	59.6	5	2	7	63.6	3	-	3	60.0
≥ 60	5	10	15	16.8	1	-	1	9.1	1	-	1	20.0
No information	-	1	1		-	-	-		-	-	-	
Mean ± SD	44.76 ± 15.10				39.45 ± 12.93				45 ± 13.81			
Education level (years) (n = 86)												
1 a 7	22	13	35	48,6	4	1	5	45.5	3	-		3	60.0
8 a 14	15	21	36	50.0	3	1	4	36.4	1	1		2	40.0
15 or more	-	1	1	1.4	2	-	2	18.1	-	-		-	-
No information	10	8	18						-	-		-	
TB presentation (n = 104)												
Pulmonary	44	40	84	95.4	9	2	11	100.0	4	1		5	100.0
Extrapulmonary	2	-	2	2.3	-	-	-		-	-		-	-
Pulmonary + Extrapulmonary	-	2	2	2.3	-	-	-		-	-		-	-
No information	1	1	2		-	-	-		-	-		-	
HIV status (n = 99)												
Negative	36	35	71	83.5	7	1	8	80.0	2	1		3	75.0
Positive	5	4	9	10.6	-	-	-	-	-	-		-	-
Not done	3	2	5	5.9	2	-	2	20.0	1	-		1	25.0
No information	3	2	5		-	1	1		1	-		1	
Comorbidities (n = 102)												
Alcohol abuse	2	1	3	3.4	-	-	-	-	-	-		-	-
Drug addiction	3	1	4	4.6	-	1	1	10.0	-	-		-	-
Smoking	9	7	16	18.4	1	-	1	10.0	-	-		-	-
Diabetes mellitus	4	6	10	11.5	1	-	1	10.0	-	-		-	-
Alcohol abuse, diabetes mellitus, drug addiction, smoking	17	6	23	26.4	2	-	2	20.0	2	-		2	40.0
None	8	14	22	25.3	4	-	4	40.0	2	1		3	60.0
Others	3	6	9	10.4	-	1	1	10.0	-	-		-	-
No information	1	2	3		1	-	1		-	-		-	
Previous TB (n = 104)												
No	20	15	35	39.8	3	2	5	45.5	-	1		1	20.0
Yes	26	27	53	60.2	6	-	6	54.5	4	-		4	80.0
No information	1	1	2		-	-	-		-	-		-	
Treatment group (n = 104)												
New case	20	15	35	39.8	3	2	5	45.5	-	1		1	20.0
Relapse	8	13	21	23.9	-	-	-	-	-	-		-	-
Retreatment	18	14	32	36.3	6	-	6	54.5	4	0		4	80.0
No information	1	1	2		-	-	-		-	-		-	
Treatment outcome (n = 92)												
Successful	29	30	59	76.6	4	1	5	50.0	2	1		3	60.0
Unsuccessful	11	7	18	23.4	4	1	5	50.0	2	-		2	40.0
No information	7	6	13		1	-	1		-	-		-	
Death (n = 98)												
No	38	35	73	88.0	5	2	7	63.6	3	1		4	80.0
Yes	6	4	10	12.0	3	-	4	36.4	1	-		1	20.0
No information	3	4	7		1	-	-		-	-		-	

TB: tuberculosis; MDR: multidrug resistant; XDR: extensively drug resistant.

We were able to collect outcome information from 92 patients, of which 77 were diagnosed with MDR-TB, ten with pre-XDR-TB and five with XDR-TB. Sixty-seven patients (72.8%) were considered ‘successfully treated’, with 62 considered cured and five completing the treatment. Twenty-five patients were found to be ‘unsuccessfully treated’, with 15 dying. The MDR-TB and pre-XDR-TB groups presented higher rates of successful outcomes (76.6% and 60%, respectively) when compared to the pre-XDR-TB group (50%) (Table IV).

Among the 106 patients included in the study, 10 patients had more than one isolate evaluated by first- and second-line drug susceptibility testing [Supplementary data **(Table II)**]. Eight of these patients exhibited resistance progression (from MDR to pre-XDR or from pre-XDR to XDR) of which three patients with MDR-TB became FQ resistant pre-XDR. Among these latter three patients, two died from TB and the other patient continued treatment after several failures and was eventually lost for follow up. As shown in Supplementary data **(Table II)**, in three patients who already presented FQ resistance, the transition from pre-XDR-TB to XDR-TB was accompanied by resistance to injectable aminoglycosides.

## DISCUSSION

The present study investigated the frequency of mutations in the main genes related to RIF, INH, FQ and aminoglycoside resistance in 156 *M. tuberculosis* isolates from 106 patients, and correlated them with phenotypic results. We also analysed the socio-demographic characteristics, treatment outcomes of these patients and the progression of drug-resistance.

Most of the isolates found to be resistant to RIF exhibited concordant results between the two methods performed. In fact, most of the mutations identified in these drug-resistant isolates were found to occur in the RRDR of *rpo*B. This is consistent with previous studies that have shown that about 95% of the mutations associated with RIF resistance occur in this region.^17^ Mutations in other segments of the *rpo*B gene have also been reported to account for approximately 5-10% of RIF resistant isolates.^17^ However, only 1.9% of the isolates evaluated in this study displayed these types of mutations and resistance phenotype. Due to the fact, RIF resistance is an MDR marker,^18^ it is of the utmost importance that efforts are made to expand the molecular analysis and/or add phenotypic susceptibility testing. An interesting finding was the identification of an isolate with an RRDR mutation and RIF susceptibility. A more detailed analysis revealed that the patient had two cultures collected, with a month interval between them. The first culture was RIF susceptible and the second was RIF resistant, thus suggesting that more than one bacterial population was present in the first sample analysed. We hypothesize that the first sample probably had two microorganism populations, with a smaller amount of the resistant population, which would explain why the isolate was susceptible in the phenotypic test and resistant in the molecular test. Further reinforcing this hypothesis is the fact the administration of RIF during the treatment period probably selected for the resistant population identified in the second isolation. In this case, the molecular test was able to detect drug resistance before the phenotypic test.

It has been reported that the Ser315Thr mutation in *kat*G and the C-15T mutation in the *fab*G/*inh*A promoter region are most frequently related to INH resistance.^5^ ^,^ ^7^ ^,^ ^19^ Indeed, we found that these were the mutations commonly found in isolates resistant to this drug. However, it should be pointed out that 21 INH resistant isolates did not possess these mutations, suggesting that other codons of the *kat*G gene or perhaps other genes, such as *inh*A, *fur*A, *kas*A, *ndh*, *fab*G and *ahp*C could also impart INH resistance.^20^ ^,^ ^21^ It is also plausible that other resistance mechanisms, such as efflux pumps, play a role in the development of this phenotype.^22^ ^,^ ^23^ Taken together these data reinforce the proposal that the best MDR-TB markers are the mutations within the *rpo*B RRDR.

Fluoroquinolones are second-line drugs essential in the treatment of RIF resistant TB and MDR-TB. According to the WHO, it is estimated that FQ resistance accounts for 20.8% of the drug-resistant TB cases,^1^ which was further corroborated by this study. When comparing our data with a previous study conducted in the same geographic region from 2011-2013,^13^ we observed a 7% decline in the number of FQ-resistant cases. It is possible that this decline is due to the implementation of the second-line drug susceptibility testing in the diagnostic routine at the IAL, which was initiated in 2012, thus improving resistance diagnosis and consequently resulting in more appropriate and effective treatment regimens.

The most commonly used target for the molecular detection of FQ resistance is the QRDR of the *gyr*A gene.^24^ In the present study, 44% of the FQ-resistant isolates harbored the Asp94Gly mutation, a value that is substantially higher than the 21-32% frequency reported in an extensive systematic review that analysed data from 18 countries in Asia, Africa, North America and Europe, but did not include Brazil.^24^ In contrast, the other mutations identified in FQ-resistant isolates in this study were present in frequencies similar to those reported in other studies.^24^ ^,^ ^25^

Importantly, when we performed the analyses of the QRDRs of both the *gyr*A and *gyr*B genes, the detection of FQ resistance was increased, which emphasises the importance of also evaluating the latter target when screening for resistance to this drug.^26^ It is also worth mentioning that eight isolates with a mutation in *gyr*B also had a polymorphism (Ser95Thr) unrelated to resistance in the *gyr*A gene. In fact, four of these isolates were from the same FQ-resistant patient who presented a previously undescribed Asp449Val mutation in the *gyr*B gene. While it is possible that this novel mutation is associated with drug resistance, additional analyses will be necessary to verify that it is involved in the resistance phenotype.

As previously mentioned, the analysis of gene fragments does not allow for the identification of all the mutations that confer drug resistance. As an alternative, it has been proposed that Whole Genome Sequencing could be employed for identifying the full range of genes associated with drug resistance as well as the presence of heteroresistance. Additionally, a recent study suggested that this approach could minimise errors in genotypic diagnoses and consequently lead to more effective treatments.^27^ ^,^ ^28^ In the isolates of this study, heteroresistance was only detected by the LPA test when the isolates were analysed in the presence of the drug. It is important to emphasise that heteroresistance was not detected in drug-free cultivation as was shown in other studies.^7^ ^,^ ^10^ ^,^ ^19^ Thus, in order to reduce the risk of misdiagnosis, it is imperative that these types of resistance tests must be performed in both the presence and absence of the drugs.

Comparing the total number of MDR-TB, pre-XDR-TB and XDR-TB patients in this study with that reported by Gallo et al.^13^ there was an observed decrease in the number of cases, demonstrating that the measures taken by the Brazilian government in 2012 likely promoted more rapid and correct TB diagnoses.

The progression of resistance in eight patients revealed that there was an evolution from MDR-TB to pre-XDR-TB due to the acquisition of resistance to FQs, an observation that is consistent with other studies.^28^ ^,^ ^29^ In these cases, treatment failure and/or treatment loss to follow up contributed to the evolution of resistance. Indeed, the misuse of FQs in the treatment of other bacterial infections, or in patients with MDR-TB, can contribute to the acquisition of resistance to this drug.^30^

While the results from the present study are consistent with previously published data, there were some limitations that deserve attention. For example, we utilised secondary data for the characteristics of patients, which may be incomplete; we only included patients with laboratory diagnosed MDR-TB in the analysis. Additionally, another potential limitation was the use of the RFLP method, which has a recognised low discriminatory power to estimate clustering rates in population studies. However, we compared the RFLP results of a same isolate grown simultaneously in media with or without drugs, thus substantially reducing the chance of misclassification. Thus, it is unlikely that either of these limitations had a significant impact on the conclusions of this work.

To the best of our knowledge, this is the first report describing the frequency of mutations related to second-line drug resistance in TB patients from the state of São Paulo, Brazil. With the exception of the novel Asp449Val mutation identified in the *gyr*B gene, the identified mutations and frequencies were consistent with previous estimates reported around the world. Furthermore, our data highlight the utility and effectiveness of combining these gene targets for the molecular diagnosis of MDR-TB, pre-XDR-TB and XDR-TB. Such insights could lead to the development of an algorithm for the rapid diagnosis of drug-resistant TB, thus resulting in better outcomes for individuals suffering from this disease.
