# Supplementary material for: Frequency of first and second-line drug resistance-associated mutations among resistant Mycobacterium tuberculosis clinical isolates from São Paulo, Brazil
Source: Mem Inst Oswaldo Cruz. 2020 May 8;115:e200055. doi: 10.1590/0074-02760200055 (PMC7207153; doi:10.1590/0074-02760200055)
Supplement: Supplementary file 1 [file 1678-8060-mioc-115-e200055-s.pdf]

TABLE I
List of primers used in this study

Target gene	Primer	Sequence (5'-3')	TM (°C)	Fragment size (bp)	Reference
rrs	*rrs*420 Forward	TTCACCATCGACGAAGGTCC	62	571	Via et al.[4]
	*rrs*990 Reverse	CTAGACGCGTCCTGTGCATGT			
	*rrs*P2	GAGTACGGCCGCAAGGCTA	66	680	Machado[6]
	rrs 1539	GGGGCGTTTTCGTGGTGCTCC			Alangaden et al.[2]
*fab*G-*inh*A	*inh*A Forward	CCTCGCTGCCCAGAAAGGGA	64	248	Gonçalves et al.[5]
	*inh*A Reverse	ATCCCCCGGTTTCCTCCGGT			
*kat*G	*kat*G Forward	GCAGATGGGGCTGATCTACG	60	555	
	*kat*G Reverse	AACTCGTCGGCCAATTCCTC			
*gyr*A	*gyr*A Forward	ATCGCCGGGTGCTCTATG	60	321	Machado[6]
	*gyr*A Reverse	GGCCGTCGTAGTTAGGGATG			
*gyr*B	*gyr*B Forward	GTCGTTGTGAACAAGGCTGTG		413	
	*gyr*B Reverse	GTGGAAATATGTTGGCCGTC			
*rpo*B	*rpo*B Forward	GGGAGCGGATGACCACCCA	60	350	Portugal et al.[3]
	*rpo*B Reverse	GCGGTACGGCGTTTCGATGAAC			

T_M: temperature of melting; bp: base pairs.

CC BY

TABLE II

Description of the treatments of patients from the State of São Paulo who progressed to more drug resistant forms of tuberculosis

ID	Gender/ Age[a]	Year of MDR-TB diagnosis	Year of pre XDR-TB diagnosis	Year of XDR-TB diagnosis	Drug resistance[b]	Mutation-*genetic target*	Treatment regimens (year, drugs received and outcome)	Outcome/ Year	Co-morbidities or risk behaviors
05	M 62	-	2012	2015	RIF, INH, PZA, EMB, CM, OFX, LFX	Asp516Val-*rpo*B, Ser315Thr-*kat*G Asp94Gly-*gyr*A A1401G-*rrs*	2010/2011: EMB, ETO, PZA, SM - Treatment failure 2011/2014: EMB, PZA, TRD, SM - Treatment failure 2014/2016: RIF, CFZ - Treatment failure 2017/2018: AM, ETO, CFZ - Treatment failure 2018: EMB, PZA, SM -Under treatment	Under treatment	None reported
18	F 41	-	2012	2013	RIF, INH, PZA, EMB, OFX, CM, AM, KN	Ser531Leu-*rpo*B, Ser315Thr-*kat*G Asp94Gly-*gyr*A A1401G-*rrs*	2010/2012: PZA, EMB, SM, TRD - Treatment failure 2012/2014: PZA, EMB, ETO, TRD, AM - Treatment failure 2014/2016: RIF, CFZ - Treatment failure	Treatment failure 2016	None reported
36	M 29	2014	2016	-	RIF, INH, PZA, EMB, LFX	Ser531Leu-*rpo*B, Ser315Thr-*kat*G Asp94Asn - *gyr*A	2014: RIF, INH, PZA, EMB - Treatment failure 2014/2015: PZA, EMB, SM, TDA - Treatment failure 2015/2016: PZA, EMB, TDA, AM - Treatment failure 2016: PZA, EMB, TDA, CFZ - Lost to follow-up 2017: PZA, EMB, CFZ - Lost to follow-up 2019: PZA, CFZ - Under treatment	Under treatment 2019	None reported
43	M 48	-	2014	2016	RIF, INH, PZA, SM, OFX, CM	Ser531Leu-*rpo*B, Ser315Thr-*kat*G Asn538Asp *gyr*B	2013/2014: EMB, TRD - Treatment failure 2014/2107: RIF, INH, CFZ - Treatment failure 2017/2019: EMB, AM, CFZ - Cured	Cured 2019	None reported
50	M 66	2013	2015	-	RIF, INH, EMB, PZA, SM, OFX	Ser531Leu-*rpo*B, Ser315Thr-*kat*G Asp94Gly *gyr*A	2013/2014: RIF, INH, PZA, EMB - Treatment failure 2014/2015: PZA, EMB, TRD - Treatment failure 2015/2017: OFX, TRD, AM, CFZ - Treatment failure 2017: PZA, EMB, TRD - Died	Died 2017	Hepatitis B and C
59	F 27	2014	2016	-	RIF, INH, EMB, PZA, OFX, LFX	Ser531Leu-*rpo*B, Ser315Thr-*kat*G Asp94Gly *gyr*A	2014/2016: PZA, EMB, TRD, AM - Treatment failure 2016: ETO, CFZ - Died	Died 2016	None reported
61	M 50	2013	-	-	RIF, INH	Ser531Leu-*rpo*B,C-15T-*inh*A	2013: RIF, INH, PZA, EMB - Treatment failure 2013/2015: PZA, EMB, ST, TRD - Cured 2017/2019: PZA, EMB, ETO - Died	Died 2019	Alcohol abuse and smoking
62	M 58	2013[c]	-	-	RIF, INH	Ser531Leu-*rpo*B, Met732Arg-*kat*G	2013: PZA, EMB, TRD - Lost to follow-up 2015: PZA, EMB, TRD, AM - Lost to follow-up 2017: RIF, INH, PZA, EMB - Treatment failure	Treatment failure 2017	None reported
72	M 36	-	2013	2016	RIF, INH, PZA, EMB, LFX, KN	Ser531Leu-*rpo*B Arg463Leu, G1430del *kat*G, A1401G *rrs*, Asp94Gly-*gyr*A	None reported	None reported	None reported
84	F 53	-	2012	2014	RIF, INH, CM, AM, KN, OFX	His526Tyr-*rpo*B, Ser315Thr-*kat*G, A1401G *rrs*, Asp449Val *gyrB*	2014/2015: ETO, CFZ - Lost to follow-up 2016: PZA, ETO, CFZ - Lost to follow-up 2018: PZA, CFZ - Under treatment	Under treatment 2018	Diabetes mel-litus, smoking

a: age; *b*: DST results at the time of XDR-TB diagnosis; *c*: patient with an isolate in 2012 only INH-resistant; INH: isoniazid; RIF: rifampicin; SM: streptomycin; PZA: pyrazinamide; EMB: ethambutol; CM: capreomycin; KN: kanamicin; AM: amikacin; OFX: ofloxacin; LFX: levofloxacin; CFZ: clofazimine; TRD: terizidone; ETO: ethionamide. F: female; M: male.
